# How are rapid diagnostic tests for infectious diseases used in clinical practice: a global survey by the International Society of Antimicrobial Chemotherapy (ISAC)

**DOI:** 10.1007/s10096-020-04031-2

**Published:** 2020-09-09

**Authors:** Stephen Poole, Jennifer Townsend, Heiman Wertheim, Stephen P. Kidd, Tobias Welte, Philipp Schuetz, Charles-Edouard Luyt, Albertus Beishuizen, Jens-Ulrik Stæhr Jensen, Juan González del Castillo, Mario Plebani, Kordo Saeed

**Affiliations:** 1 NIHR Southampton Biomedical Research Centre, University Hospital Southampton NHS Foundation Trust, Southampton, UK; 2 The Johns Hopkins University School of Medicine, Baltimore, MD USA; 3 Department of Medical Microbiology and Radboudumc Center for Infectious Diseases, Radboudumc, Nijmegen, Netherlands; 4 Hampshire Hospitals NHS Foundation Trust, Basingstoke, UK; 5 Department of Respiratory Medicine and member of the German Centre of Lung Research, Medizinische Hochschule, Hannover, Germany; 6 Internal Medicine and Emergency Medicine Endocrinology, Diabetes & Clinical Nutrition Medical University, Department Kantonsspital Aarau, Tellstrasse CH, -5001 Aarau, Switzerland; 7 Service de Médecine Intensive Réanimation, Institut de Cardiologie, Groupe Hospitalier Pitié-Salpêtrière, Sorbonne Université, Assistance Publique Hôpitaux de Paris, Paris, France; 8 Intensive Care Center, Medisch Spectrum Twente, Enschede, Netherlands; 9 Department of Internal Medicine, Respiratory Medicine Section, Herlev-Gentofte Hospital, Kildegaardsvej 28, 2900 Hellerup, Denmark; 10 Department of Clinical Medicine, Faculty of Health Sciences, University of Copenhagen, Blegdamsvej 3B, 2200 Copenhagen, Denmark; 11 Emergency Department, Hospital Clínico San Carlos, Madrid, Spain; 12 School of Medicine and Surgery, University of Padova, & Centre of Biomedical Research, Vento Region, Padova, Italy; 13 School of Medicine, University of Southampton, Southampton, UK; 14 Microbiology Innovation and Research Unit (MIRU), Microbiology Department, Southampton University Hospitals NHS Foundation Trust, Southampton, SO16 6YD UK

**Keywords:** Rapid diagnosis, Infection, Microbiology, Clinical governance, Point of care, POCT

## Abstract

Novel rapid diagnostic tests (RDTs) offer huge potential to optimise clinical care and improve patient outcomes. In this study, we aim to assess the current patterns of use around the world, identify issues for successful implementation and suggest best practice advice on how to introduce new tests. An electronic survey was devised by the International Society of Antimicrobial Chemotherapy (ISAC) Rapid Diagnostics and Biomarkers working group focussing on the availability, structure and impact of RDTs around the world. It was circulated to ISAC members in December 2019. Results were collated according to the UN human development index (HDI). 81 responses were gathered from 31 different countries. 84% of institutions reported the availability of any test 24/7. In more developed countries, this was more for respiratory viruses, whereas in high and medium/low developed countries, it was for HIV and viral hepatitis. Only 37% of those carrying out rapid tests measured the impact. There is no ‘one-size fits all’ solution to RDTs: the requirements must be tailored to the healthcare setting in which they are deployed and there are many factors that should be considered prior to this.

**Electronic supplementary material:**

The online version of this article (10.1007/s10096-020-04031-2) contains supplementary material, which is available to authorized users.

## Introduction

Rapid diagnostic tests (RDTs) are increasingly used in clinical practice to provide actionable information for patient care in a timely manner, ideally at the time and location of the patient’s interaction with health care systems. RDTs (often referred to as point-of-care tests (POCT) when deployed near-patient) are often simple to use and therefore can offer diagnostic support in resource-limited settings or away from more sophisticated diagnostic laboratory support, for example in primary care.

The treatment of many infectious diseases is time-critical. A test that facilitates early-directed therapy increases the chance of good patient outcomes and promotes good antimicrobial stewardship. Furthermore, the early identification of highly transmissible illnesses allows healthcare services in high-income countries to rapidly isolate patients and limit the spread of disease: a benefit which has been particularly highlighted with the emergence of SARS-CoV-2.

The last decade has seen a boom in rapid diagnostic products, with many developed and approved by healthcare authorities around the world [1] for a variety of different infections including gastroenteritis [2], bloodstream infections [3], pneumonia [4] and respiratory viruses [5]. Formats of these tests include lateral flow assays and polymerase chain reaction (PCR).

There are potential pitfalls around the implementation of RDTs. Many are expensive, and robust evidence for tangible clinical benefit to justify this outlay can be lacking. For some, sensitivity and specificity may be lower than established laboratory tests and therefore require that these can only be applicable to specific situations (e.g. when the pre-test probability is high). Governance, quality control and assurance can be challenging, particularly when RDTs are not sited within a traditional laboratory setup. These challenges differ around the world depending on local health diagnostic regulations, availability of resource, local epidemiology and patient expectation.

The International Society of Antimicrobial Chemotherapy (ISAC)’s Rapid Diagnostics and Biomarkers Working Group conducted this international survey aiming to identify and highlight some key issues related to RDTs and their impacts in clinical practice and provide a number of key points to consider while adopting a RDT.

## Methods

A questionnaire (Survey Monkey®) was devised and approved by the Working Group (supplementary materials). The survey included 9 questions about the experience of RDTs:

1. What RDTs are available in your setting 24/7?
2. Any others not listed?
3. If you do not have RDTs, what are the barriers to getting them in your institution?
4. Who performs the RDTs?
5. How are results communicated?
6. Do you measure the impact of the tests?
7. How are these measured (if applicable)?
8. Who is responsible for governance related issues and quality controls of RDTs and results?
9. Do you have any recommendations for when and what rapid diagnostic test should be available in your setting? Or do you want to share any impact on your rapid diagnostics tests?

The questionnaire was circulated to 400 ISAC members via ISAC secretaries and respondents were given 4 weeks to respond during December 2019 and January 2020 with at least two reminders. Everyone surveyed was either in a position to request or deliver tests. Among the questions, there was an optional question asking responders to provide their specific role and institution.

The location of institutions was linked to a United Nations (UN) human development index (HDI) ranking (very high, high, medium or low) [6]. This is a widely used, blunt representation of a nation’s development which considers life expectancy, income per capita and education.

## Results

**Fig. 1:**
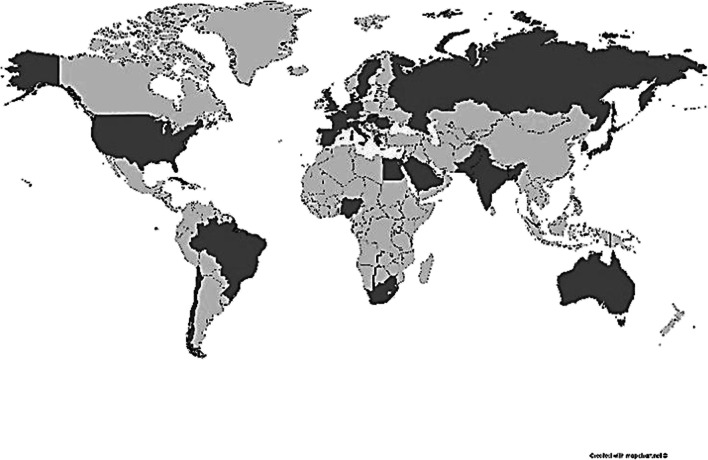
Resident countries of specialists responding to survey (dark grey)

Responses were received from 81 ISAC members representing 31 countries (Fig. 1). This represented 20% of those initially surveyed. Six respondents did not disclose their nationality. 81% of those who did disclose their nationality were received from countries classified as very highly developed on the UN HDI, 11% were from highly developed nations and the remaining 8% from countries classified as having medium or low levels of development.

**Fig. 2:**
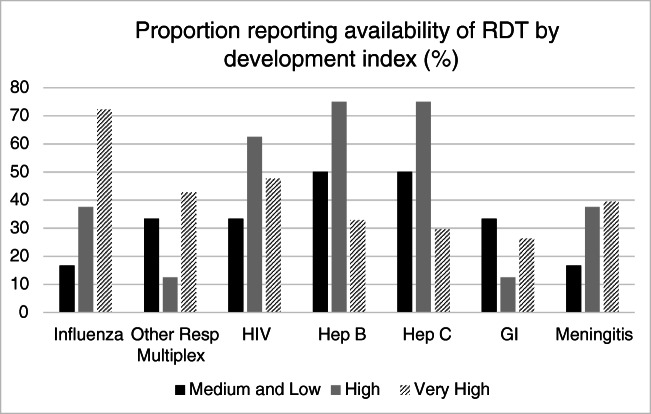
Proportion of availability of RDT by development index. Other resp multiplex, other respiratory multiplex; Hep, hepatitis; GI, gastrointestinal tests

**Fig. 3:**
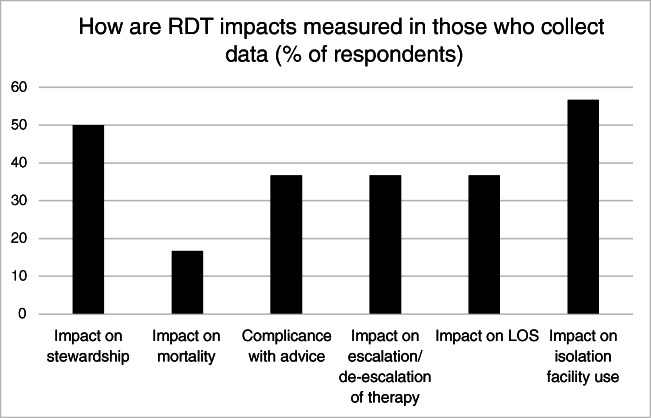
How are impacts of RDTs measured. LOS, length of stay

13/81 (16%) respondents reported no available RDTs. The proportion of those who have these available is reported in Fig. 2. The main barrier reported for not adopting RDTs was financial (64%), and other reasons were a lack of expertise (6%) or lack of applicability to their clinical setting (6%). 4% cited a lack of interest in the tests. Only 37% of those with RDTs reported measuring the impacts of their tests in any way (Fig. 3).

**Fig. 4:**
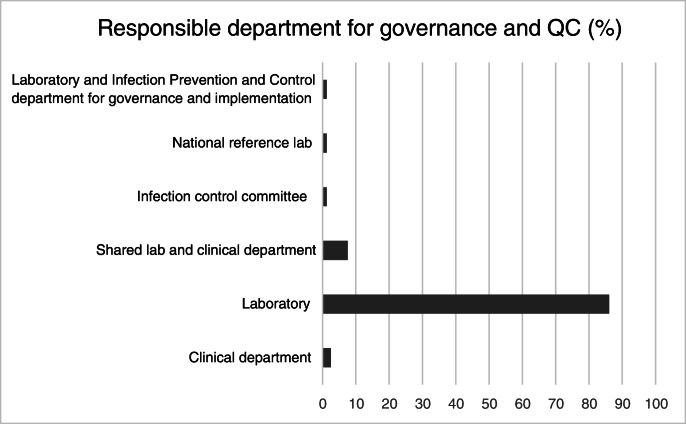
Who is responsible for governance and quality control of RDTs in your institution?

91% of those with RDTs reported the laboratory carrying out the test. 28% reported the emergency department performing them. Other clinical settings rarely carried out the tests (5% in clinics and 7% inwards). The governance structure for RDTs is presented in Fig. 4.

The most common way for reporting was in the electronic patient file (51%); fewer institutions generate the report in real time (36%). 47% of institutions directly phone the result to the requesting clinician. One respondent reported still generating paper reports, one reporting by email, one by SMS and one not generating any specific laboratory reports as the test is done in the assessment area.

## Discussion

The survey has given us an insight into what is happening globally with RDTs. Many respondents reported 24/7 availability of tests. Very high-income countries had higher proportional availability of rapid influenza and respiratory virus tests. In lower-income countries, however, a lower proportion of respondents reported the availability of these tests, but HIV and hepatitis testing were available in greater proportions. The explanation for this pattern is likely multifactorial. In general, the epidemiology of chronic viral hepatitis and HIV is such that they are more prevalent in developing countries where public health interventions are less likely to identify and treat patients early in the course of illness [7]. The priorities for treatment are also different: influenza management in secondary care is a less pressing need in resource-restricted settings where patient isolation facilities are less readily available. Furthermore, the clinical impact relative to the cost of identifying a case of influenza is less than HIV or viral hepatitis where early identification and treatment make a greater difference [8, 9]. The relative cost of each test is likely to also be a factor in the difference of availability, with multiplexed assays generally being considerably more expensive and requiring more complex logistical support. Methods for reducing the costs of many RDTs are lacking, which limit their availability in low-income settings.

There are still major gaps in capturing the impact of RDTs on decision making in a systematic manner. Only 37% of users measure impact. 64% of those surveyed reported that lack of money was the major barrier to bringing in RDTs in their institution. Developing robust impact recording systems, such as regular audit cycles, coupled with cost-effectiveness analyses are crucial to support business cases for new RDTs.

The current setup of RDTs appears to be more laboratory centred: governance and quality control are the responsibility of laboratories in the vast majority of those surveyed. 90% of those who responded to the survey said tests were carried out in their institution by laboratory staff. Simpler tests lend themselves more towards near-patient deployment and a CLIA waiver is often a good indicator of this. While there are a number of existing international regulatory processes for drugs and medications, providing safeguards for their safety and efficacy, they are often lacking for RDTs [10, 11]. As a result, diagnostic tests are often sold and used in the developing world without any evidence of effectiveness. For example, Mak et al. [12] reported the sensitivity of an RDT for SARS-CoV-2 of 11.1–45.7% when the manufacturer had claimed it was 98%.

The benefit of RDTs can be lost if not coupled with rapid pre- and post-analytical phases. The survey identified that less than half of the results are communicated to the requester directly, and only 35% of reports are generated in real-time on computers. This means delays are introduced as clinicians look up results. Interestingly in some institutions, results are sent out by SMS or email to requesting clinicians which would optimise the reporting process. Identification of certain infectious organisms may have wider public health implications, for example, *Legionella*; therefore we advocate real-time connection for these results to systems that allow rapid reporting to responsible public health authorities.

A limitation to the method we should consider is the selection bias towards ISAC members who would be motivated to respond to the survey: potentially those who have the greatest interest in RDTs or who are highly critical of them. There is also a bias towards respondents with greater resources suggested by the fact that at least 90% of tests had a laboratory involvement. Furthermore, the survey size is relatively small and certain world regions (especially Southeast Asian nations and Sub-Saharan African nations) are poorly represented.

The main aims of RDTs are to improve patient care most efficiently within well-managed healthcare systems. We therefore suggest a number of best practices for implementation of RDTs (Table 1).

**Table 1:** Best practices for RDT implementation

Clinical scenario	Identify what scenario the test will impact. Assess the number of patients that the test will impact per year.
Test requirements	Determine relevant patient outcome(s) for measuring impact. Consider what turnaround time can usefully influence clinical decision making to achieve tangible improvements in this outcome(s). Ascertain the acceptable sensitivity and specificity, after taking account for likely pre-test probability of disease. Identify a suitable source of funding, and consider ongoing financial requirements for support and reagents.
Logistics and reporting	Decide on siting of RDT (laboratory vs POCT). Provide rapid reporting method which integrates with existing reporting mechanisms. Explore need for clinical specialist reporting or result interpretation. If wider public health consideration of RDT target organism(s), ensure results can be readily compiled for appropriate agencies (e.g. influenza or *Legionella* reporting). Consider need for material for additional studies, such as confirmatory testing, internal validation, laboratory research and development, or strain characterization.
Quality control and Governance	Decide responsible body governance body. Identify source of suitable QC materials (particular consideration in highly multiplexed RDTs). Instigate regular internal quality assurance programme. Set up external quality assurance programme, preferably with inter-laboratory comparison. Achieve and maintain reliable technical competency with the RDT. Set up regular audit cycles which capture RDT benefit.

## Conclusion

For RDTs there is no ‘one-size-fits-all’ model; modelling of tests and costs are wildly different for different healthcare systems. Our survey highlights the availability of these tests in different resource settings, as well as the current models for governance, quality control and reporting.

## Electronic supplementary material

ESM 1 (DOCX 27 kb)
