# Supplementary material for: How are rapid diagnostic tests for infectious diseases used in clinical practice: a global survey by the International Society of Antimicrobial Chemotherapy (ISAC)
Source: Eur J Clin Microbiol Infect Dis. 2020 Sep 9;40(2):429–34. doi: 10.1007/s10096-020-04031-2 (PMC7478941; doi:10.1007/s10096-020-04031-2)
Supplement: Supplementary file 1 — (DOCX 27 kb) [file 10096_2020_4031_MOESM1_ESM.docx]

## How are Rapid Diagnostic Tests for Infectious Diseases used in clinical practice; a global survey by the International Society of Antimicrobial Chemotherapy (ISAC)

Stephen Poole^1^, Jennifer Townsend^2^, Heiman Wertheim^3^, Stephen P Kidd^4^, Tobias Welte^5^, Philipp Schuetz^6^, Charles-Edouard Luyt^7^, Albertus Beishuizen^8^, Jens-Ulrik Stæhr Jensen^9, 10^, Juan González del Castillo^11^, Mario Plebani^12^, Kordo Saeed^13,14^*

**Supplementary material**

**Appendix 1: Table of list of questions on the original questionnaire**

| **Number** | **Question** | **Options** |
| --- | --- | --- |
| 1 | What rapid infection diagnostics testing do you do in your setting 24/7 with results available within 2 hours directly from patient's specimen? | 1. Influenza 2. A range of respiratory tests including influenza 3. HIV 4. Hepatitis B 5. Hepatitis C 6. Gastroenteritis panel 7. Meningoencephalitis 8. Others: please state |
| 2 | Do you have other "rapid infection diagnostic testing" that does not fit the above criteria? Not include above? | 1. Yes (please state) 2. No |
| 3 | If you do not have rapid infection diagnostic testing, what are the barriers? (Please tick all the appropriate) | 1. Financial 2. Expertise 3. Not applicable to our setting e.g. lack of impact 4. Lack of interest 5. Other: please specify |
| 4 | Who is performing (doing) the rapid infection diagnostic test(s)? | 1. Emergency department 2. Wards 3. Clinics 4. Laboratory staff 5. Combination as depends on test 6. Other: please specify |
| 5 | How do you communicate rapid infection diagnostic testing results? | 1. Available on the computer in real time 2. Available on the computer when a report is generated by the laboratory 3. Phone results to requesters 4. Other: please specify |
| 6 | Do you measure impact of rapid infection diagnostic testing? | 1. Yes 2. No |
| 7 | If yes to Q6 what impact do you measure | 1. Do not measure impact 2. Impact on antibiotic stewardship 3. Compliance with advice 4. Impact on mortality 5. Impact on length of stay 6. Impact on escalation or de-escalation of therapy 7. Impact on infection prevention (e.g. isolation, freeing up isolation rooms) 8. Other: please specify |
| 8 | Who is responsible for governance related issues and quality controls of rapid diagnostic tests and results? | 1. Laboratory 2. Clinical department 3. Other: please specify |
| 9 | Do you have any recommendations for when and what rapid diagnostic test should be available in your setting? Or do you want to share any impact on your rapid diagnostics tests? | 1. (Please list) |

**Appendix 2: Additional free text responses for questions**

| **Question number/ response option** | **Responses (number of responses greater than one)** |
| --- | --- |
| 1/ others: please state | - Blood PCR multiplex panel - CPEs - Clostridioides difficile (3) - Multiplex assay only for specific populations - Galactomannan Aspergillus antigen - Hepatitis E - Streptococcus pyogenes (2) - Dengue - Legionella and pneumococcal urinary antigen (2) - Cryptococcus (2) - Leptospirosis - Malaria (3) - MRSA (4) - RSV - STD urine |
| 2/ yes (please state) | - Batched biofire - Norovirus, other serology eg VZV, can do MRSA and TB PCR - C. difficile (4) - Crypto(coccus), Aspergillus - For blood stream infections - Galactomannan for aspergillus; NAAT GC and Chlamydia - Gene Xpert MTB Rif R; Carba; VRE; MRSA; Norovirus; - Legionella. MRSA (seven days a week but not at night) - Malaria - MRSA active surveillance screening - PCR CCHF, Dengue, Chikungunya, Zika, Influenza, Leishmaniasis - PCT - RSV - RSV POC testing - RSV, Rota/adeno virus test - RSV/Influenza rapid PCR - STI - Urine species and susceptibility testing |
| 3/ other: please specify | - Fairly fast lab testing from central lab - mostly <2 h - Place to hold patients while pending results e.g. flu - Public health labs at NIH have rapid diagnostic testing - Staffing (2) - Unclear of the benefits in children in real world settings |
| 4/ other: please specify | N/A |
| 5/ other: please specify | - Done in clinical setting - SMS - Email requester - Written report |
| 7/ other: please specify | N/A |
| 8/ other: please specify | N/A |

**Appendix 3: Responses to question 9**

Do you have any recommendations for when and what rapid diagnostic test should be available in your setting? Or do you want to share any impact on your rapid diagnostics tests?

What diagnostic tests should be available in your setting and when?

- CAP with admission
- Carbapenem resistant bacteria for high risk patients to aid in early isolation (6)
- Emergency department
- MRSA (2)
- If BioFire counts as RDT than this is something I seek :)
- We have all the necessary ones
- If laboratory pending time went up, I would want POC / PON tests
- Influenza (2)
- Respiratory multiplex
- it is highly dependent of the institution, its organization (single hospitals vs multiple hospitals with only one central lab), distance between lab and hospitals, length of opening period of the lab, money, profile of recruitment, ...
- Meningitis multiplex panel (4)
- norovirus should both be available 24/7
- TB (2)
- Gastroenteritis
- Urine testing should be available as antibiotic administration is high for UTI, important antimicrobial stewardship issue

Do you want to share any impact of your RDTs?

- Our rapid flu test has not had much impact because patients with IFI still need to have droplet precautions regardless of the result
- Anecdotally, we have seen impact on LOS and de-escalation of antibiotic therapy
- Impact of rapid diagnostic tests for tropical infections like Dengue/Malaria
- We usually use RDT results during ward rounds to enable clinical teams to de-escalate therapy and have found it a very useful strategy.

Other comments

- Main issue is adequate storage and data recording, analysis and sharing
- 24/7 availability is utopia at present
- All lab staff should be trained to use equipment related to the testing of these sample types. not all tests should be available on the wards as the lab will always be responsible for the results and ward staff may not be fully trained to trouble shoot any issues that may arise. the rapid diagnostic test should require a minimum level of training for busy ward staff.
- Protocols to measure the impact of rapid tests are valuable for clinical settings.
- Rapid diagnostic test should be available in the majority of hospitals
- The role of rapid testing is unclear in children. Paucity of evidence demonstrating an impact on clinical outcome of antimicrobial use. Not keen on introducing more rapid testing for resp tract infections in children due to the opportunity cost (time, financial) in absence of evidence of clinical benefit.
- We have also reviewed other tests eg cognitor from Momentum which are working on rapid bacteraemia detection or exclusion. this could help with antimicrobial stewardship
- We would like to have the rapid tests used widely.
